# Analysis of Errors in Dictated Clinical Documents Assisted by Speech Recognition Software and Professional Transcriptionists

**DOI:** 10.1001/jamanetworkopen.2018.0530

**Published:** 2018-07-06

**Authors:** Li Zhou, Suzanne V. Blackley, Leigh Kowalski, Raymond Doan, Warren W. Acker, Adam B. Landman, Evgeni Kontrient, David Mack, Marie Meteer, David W. Bates, Foster R. Goss

**Affiliations:** 1 Department of Medicine, Brigham and Women’s Hospital, Boston, Massachusetts; 2 Harvard Medical School, Boston, Massachusetts; 3 Department of Information Systems, Partners HealthCare, Boston, Massachusetts; 4 Geisinger Commonwealth School of Medicine, Scranton, Pennsylvania; 5 Department of Emergency Medicine, Brigham and Women’s Hospital, Boston, Massachusetts; 6 Department of Hospital Medicine, North Shore Medical Center, Salem, Massachusetts; 7 University of Colorado School of Medicine, Aurora; 8 Department of Computer Science, Brandeis University, Waltham, Massachusetts; 9 Department of Emergency Medicine, University of Colorado, Aurora

## Abstract

**Importance:**

Accurate clinical documentation is critical to health care quality and safety. Dictation services supported by speech recognition (SR) technology and professional medical transcriptionists are widely used by US clinicians. However, the quality of SR-assisted documentation has not been thoroughly studied.

**Objective:**

To identify and analyze errors at each stage of the SR-assisted dictation process.

**Design, Setting, and Participants:**

This cross-sectional study collected a stratified random sample of 217 notes (83 office notes, 75 discharge summaries, and 59 operative notes) dictated by 144 physicians between January 1 and December 31, 2016, at 2 health care organizations using Dragon Medical 360 | eScription (Nuance). Errors were annotated in the SR engine–generated document (SR), the medical transcriptionist–edited document (MT), and the physician’s signed note (SN). Each document was compared with a criterion standard created from the original audio recordings and medical record review.

**Main Outcomes and Measures:**

Error rate; mean errors per document; error frequency by general type (eg, deletion), semantic type (eg, medication), and clinical significance; and variations by physician characteristics, note type, and institution.

**Results:**

Among the 217 notes, there were 144 unique dictating physicians: 44 female (30.6%) and 10 unknown sex (6.9%). Mean (SD) physician age was 52 (12.5) years (median [range] age, 54 [28-80] years). Among 121 physicians for whom specialty information was available (84.0%), 35 specialties were represented, including 45 surgeons (37.2%), 30 internists (24.8%), and 46 others (38.0%). The error rate in SR notes was 7.4% (ie, 7.4 errors per 100 words). It decreased to 0.4% after transcriptionist review and 0.3% in SNs. Overall, 96.3% of SR notes, 58.1% of MT notes, and 42.4% of SNs contained errors. Deletions were most common (34.7%), then insertions (27.0%). Among errors at the SR, MT, and SN stages, 15.8%, 26.9%, and 25.9%, respectively, involved clinical information, and 5.7%, 8.9%, and 6.4%, respectively, were clinically significant. Discharge summaries had higher mean SR error rates than other types (8.9% vs 6.6%; difference, 2.3%; 95% CI, 1.0%-3.6%; *P* < .001). Surgeons’ SR notes had lower mean error rates than other physicians’ (6.0% vs 8.1%; difference, 2.2%; 95% CI, 0.8%-3.5%; *P* = .002). One institution had a higher mean SR error rate (7.6% vs 6.6%; difference, 1.0%; 95% CI, −0.2% to 2.8%; *P* = .10) but lower mean MT and SN error rates (0.3% vs 0.7%; difference, −0.3%; 95% CI, −0.63% to −0.04%; *P* = .03 and 0.2% vs 0.6%; difference, −0.4%; 95% CI, −0.7% to −0.2%; *P* = .003).

**Conclusions and Relevance:**

Seven in 100 words in SR-generated documents contain errors; many errors involve clinical information. That most errors are corrected before notes are signed demonstrates the importance of manual review, quality assurance, and auditing.

## Key Points

**Question:**

How accurate are dictated clinical documents created by speech recognition software, edited by professional medical transcriptionists, and reviewed and signed by physicians?

**Findings:**

Among 217 clinical notes randomly selected from 2 health care organizations, the error rate was 7.4% in the version generated by speech recognition software, 0.4% after transcriptionist review, and 0.3% in the final version signed by physicians. Among the errors at each stage, 15.8%, 26.9%, and 25.9% involved clinical information, and 5.7%, 8.9%, and 6.4% were clinically significant, respectively.

**Meaning:**

An observed error rate of more than 7% in speech recognition–generated clinical documents demonstrates the importance of manual editing and review.

## Teaser

This study characterizes error frequency and type in clinical documents from 2 health systems signed off on by attending physicians after having been generated using speech recognition software and having been edited by medical transcriptionists.

## Introduction

Clinical documentation is among the most time-consuming and costly aspects of using an electronic health record (EHR) system.^1,2^ Speech recognition (SR), the automatic translation of voice into text, has been a promising technology for clinical documentation since the 1980s. A recent study reported that 90% of hospitals plan to expand their use of SR technology.^3^ There are 2 primary ways that SR can assist the clinical documentation process. In this study, we evaluated back-end SR (Figure, A), in which physicians’ dictations are captured and converted to text by an SR engine. The SR-generated text is edited by a professional medical transcriptionist (MT), then sent back to the physician for review. The other type is commonly called *front-end SR* (Figure, B). Here, physicians dictate directly into free-text fields of the EHR and edit the transcription before saving the document.

**Figure.**
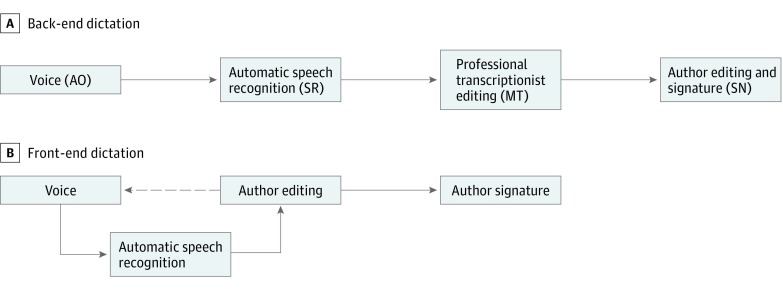
Stages of Back-End and Front-End Dictation There are two 2 primary ways that speech recognition (SR) can assist the clinical documentation process. In back-end SR, clinicians’ dictations, the audio original (AO), are captured and converted to text by an SR engine. The SR-generated text is edited by a professional medical transcriptionist (MT), then sent back to the clinician for review and a signed note (SN). In front-end SR, clinicians dictate directly into free-text fields of the electronic health record and edit the transcription.

A recent study in Australia evaluated the type and prevalence of errors in documents created using a front-end SR system and those created using a keyboard and mouse.^4^ A higher prevalence of errors was found in notes created with SR, both overall and across most error types included in the analysis. While front-end SR is becoming more widely used, back-end SR systems remain in use in many health care institutions in the United States, and significant productivity enhancements associated with these systems have been demonstrated.^5,6,7^ However, to our knowledge, the quality and accuracy of clinical documents created using back-end SR have not been thoroughly studied.

Medical errors largely result from failed communication.^8^ Clinical documentation is essential for communication of a patient’s diagnosis and treatment and for care coordination between clinicians. Documentation errors can put patients at significant risk of harm.^9^ An analysis of medical malpractice cases found that incorrect information (eg, faulty data entry) was the top EHR-related contributing factor, contributing to 20% of reviewed cases.^10,11^ It is therefore in the best interest of both patients and clinicians that medical documents be accurate, complete, legible, and readily accessible for the purposes of patient safety, health care delivery, billing, audit, and possible litigation proceedings.^12,13,14,15^ As more medical institutions adopt SR software, we need to better understand how it can be used safely and efficiently.

In this study, we analyzed errors at different processing stages of clinical documents collected from 2 institutions using the same back-end SR system. We hypothesized that error rates would be highest in original SR transcriptions, lower in notes edited by transcriptionists, and lower still in physicians’ signed notes (SN). We also expected significant differences in mean error rates between note types and between notes created by physicians of different specialties. We expected no significant difference in mean error rates among physicians of different sexes or from different institutions.

## Methods

This cross-sectional study was conducted and reported following the Strengthening the Reporting of Observational Studies in Epidemiology (STROBE) reporting guideline.^16^ Approval for this study was obtained from the Partners Human Research Committee and the Colorado Multiple Institutional Review Board. The study was determined by both institutional review boards to meet the criteria for a waiver of informed consent. Analysis was conducted from June 15, 2016, to November 17, 2017.

### Clinical Setting and Data Collection

This study used 217 documents dictated between January 1 and December 31, 2016, from hospitals at 2 health care organizations: Partners HealthCare System in Boston, Massachusetts, and University of Colorado Health System (UCHealth) in Aurora, Colorado. Both organizations use Dragon Medical 360 | eScription (Nuance). Because hospitals use dictation for different note types, we collected a stratified random sample based on the different note types dictated at each hospital. The sample includes 44 operative notes, 83 office notes, and 40 discharge summaries from Partners HealthCare and 15 operative notes and 35 discharge summaries from UCHealth. We collected data for dictating physicians’ age, sex, and specialty.

We reviewed each note at the 4 main processing stages of dictation. This included (1) our own transcription of the original audio recording (used as the criterion standard, described in the following section), (2) the note generated by the SR engine of the vendor transcription service (SR note), (3) the note following revision by a professional MT (MT note), and (4) the note after having been reviewed and signed by the physician (SN).

### Criterion Standard, Annotation Schema, and Annotation Process

To create the criterion standard for each note, a PharmD candidate or medical student, under the supervision of 2 practicing physicians, created a transcription of the note while listening to the original audio and using the MT note as a reference. The audio was played repeatedly, at different speeds, to ensure the transcription’s accuracy. Medical record review was conducted to validate notes’ content, such as by referring to a patient’s structured medication list to verify a medication order that was partially inaudible in the original audio recording.

A team of clinical informaticians, computational linguists, and clinicians developed a schema for identifying and classifying errors iteratively over multiple annotation rounds. The schema includes 12 general types (eg, insertion), 14 semantic types (eg, medication), and a binary classification of clinical significance.^17^ An error was considered clinically significant if it could plausibly change a note’s interpretation, thereby potentially affecting a patient’s future care either directly (eg, by influencing clinical decisions or treatment options) or indirectly (eg, by causing billing errors or affecting litigation proceedings). The complete annotation schema is shown in Table 1. The schema includes brief descriptions of each type of error. Each error type is also accompanied by 1 or more examples found during the course of our annotation.

**Table 1.** Annotation Schema

Error Type	Description	Examples	
General type			
Insertion	One or more words was added to the transcription	AO: There is distal biliary obstruction observed	
		SR: There is no distal biliary obstruction observed	
Deletion	One or more words was deleted from the transcription	AO: CHADS2 VASC score 4	
		SR: score	
Substitution			
Enunciation	An error due to a mispronunciation or failure to enunciate by the speaker	AO: to find a homeopathic provider	
		SR: defined homeopathic provider	
Suffix	The root word is correct, but there is an incorrect, added, or omitted suffix	AO: mental status worsened	
		SR: mental status worsens	
Prefix	The root word is correct, but there is an incorrect, added, or omitted prefix	AO: Inadequate evaluation to exclude neoplasia	
		SR: Adequate evaluation to exclude neoplasia	
Spelling	The transcriptionist made a spelling error when editing the output of the SR system	AO: we counseled him on risk of infection	
		MT: we counseled hom on risk of infection	
Homophone	One word has been substituted for another identically pronounced word	AO: serial high resolution anoscopy	
		SR: cereal high resolution anoscopy	
Dictionary	An error likely due to the target word not being present in the SR system’s dictionary	AO: driving a Camry and hit another car	
		SR: driving an Academy and hit another car	
Nonsense	A substitution that is so far off that it is unclear which category (if any) it falls under	AO: follow up in 3 to 5 d	
		SR: neck veins are evaluated	
No.^a^	Any error involving a number, whether it is written as a digit (2) or as a word (two)	AO: the patient is a 17-year-old female	
		SR: the patient is a 70-year-old female	
Punctuation^b^	A period, comma, or other punctuation mark was present where it should not have been	AO: at discharge she had no flank tenderness	
		SR: at discharge. She had no flank tenderness	
Semantic type			
General English	Any English words that do not fit into the categories below	AO: which she would otherwise forget	
		SR: which she would otherwise for gas that	
Stop word	Common English words^17^	AO: intermittent pain under the right breast	
		SR: intermittent pain in the right breast	
Medication	Medication names and dose information	AO: initiated on lamotrigine therapy	
		SR: initiated on layman will try therapy	
Diagnosis	Any words that are part of a specific medical diagnosis	AO: Dengue	
		SR: DKA [diabetic ketoacidosis]	
Laboratory test	Laboratory test names and results	AO: TSH of 26.7	
		SR: TSH of 22nd 6.7	
Imaging test	Imaging examination names and types and examination results	AO: nonobstructive on CT imaging	
		SR: nonobstructive on imaging	
Procedure	Procedure names and descriptions	AO: ligament was released on the leading edge	
		SR: ligament was released operating edge	
Physical examination	Any information directly related to the physical examination and any associated values	AO: T 36.7 degrees	
		SR: T3-T7 disease	
Patient or physician identifier	Any words involving patient or physician metadata (names, MRN, etc)	AO: SURGEON: [surgeon’s real name]	
		SR: SURGEON: Stathis stairs	
Date	Any dates, written with words (January 1, 2017) or with numbers (01/01/2017)	AO: 10/10/2016	
		SR: 10/10/2000	
Symptom	Any symptom or description of symptoms	AO: very mild arthralgias	
		SR: very also arthralgias	
???	When ??__?? (or similar) is left in the note, or when something is complete nonsense	AO: no foreign material was identified	
		MT: no foreign ??__?? was identified	
Clinical significance	Any error that could plausibly change a note’s interpretation, thereby giving it the potential to affect a patient’s future care	1	AO: Long-term anticoagulation should be discussed with her PCP
			SR: Long-term she should be discussed with her PCP
		2	AO: Rapamune 4 mg po daily
			SR: Verapamil 4 mg po daily
		3	AO: The patient disagreed with this recommendation
			SR: The patient did agree with this recommendation
		4	AO: Inadequate evaluation to exclude neoplasia
			SR: Adequate evaluation to exclude neoplasia
		5	AO: Allergies: Furosemide, gabapentin, oxcarbazepine, yellow dye
			SR: Allergies: Yellow dye
		6	AO: There is distal biliary obstruction observed
			SR: There is no distal biliary obstruction observed
		7	AO: Oxycodone 5 mg oral q4h PRN for pain
			SR, MT, SN: Oxycodone 5 mg oral q4h for pain

Abbreviations: AO, audio original (ie, criterion standard); MRN, medical record number; MT, medical transcriptionist-edited notes; SN, signed notes; SR, speech recognition.

^a^ A number error is a more specific type of insertion, deletion, or substitution.

^b^ A punctuation error is a more specific type of insertion or substitution.

We used Knowtator,^18^ an open-source annotation tool, to annotate notes at each stage. Two annotators (1 computational linguist and 1 medical student) independently annotated the SR-transcribed, transcriptionist-edited, and signed versions of each note for errors. Each document was further annotated for the presence or absence the following changes: automatic abbreviation expansion by the SR system, disfluencies or misspoken words on the part of the dictating physician, stylistic changes (eg, rewording a grammatically incorrect sentence) by the transcriptionist and the signing physician, rearranging of the note’s content by the transcriptionist and the physician, and the addition and removal of content by the physician prior to signing.

Two practicing physicians independently evaluated errors for clinical significance, and disagreements were reconciled through discussion.

### Measures

We determined the time required to dictate a note, along with each note’s turnaround time and clinician review time. We defined turnaround time as the length of time between the original dictation’s completion and when the transcriptionist-revised document was sent back to the EHR. Physician review time was the length of time between when the transcription was returned to the physician and when the physician signed the note.

For each version of each note, we analyzed the differences between that note and the corresponding criterion standard note. We determined the error rate (ie, the number of errors per 100 words), the median error rate with interquartile ranges, the mean number of errors per note, the frequency of each error type (the number of errors of a specific type divided by the total number of errors), and the percentage of notes containing at least 1 error. We conducted these analyses for all errors and for just those errors that were found to be clinically significant. Throughout our analyses, a document’s error rate is defined as the total number of errors it contains (or, equivalently, the total number of insertions, deletions, and substitutions) divided by the number of words in the corresponding criterion standard.

We calculated interannotator agreement using a randomly selected subset of 33 notes, which included 7 SR-transcribed notes and 26 transcriptionist-edited notes, considering these stages’ variations in error complexity (eg, transcriptionists’ edits often involve subtle rewordings, which must be distinguished from true errors). Agreement was defined as the percentage of errors for which both annotators selected the same general and semantic type. For each error, we required only that the spans selected by each annotator overlap with one another to some degree, rather than requiring exact span matches. For clinical significance, agreement was defined as the percentage overlap between the 2 physicians’ classifications.

### Statistical Analysis

Analyses were conducted in R statistical software (R Project for Statistical Computing)^19^ with *t* tests used to identify significant differences in mean error rates at each stage by sex, specialty, and note type. For comparisons involving more than 2 groups (eg, specialty), each group’s mean error rate was compared with that of all other groups combined. We calculated the Pearson correlation coefficient (*r*) to measure the strength of associations between error rate and physician age and between error rate and document length. We considered 2-sided *P* values of less than .05 to be statistically significant.

## Results

Among the 217 notes, there were 144 unique dictating physicians: 44 female (30.6%) and 10 unknown sex (6.9%). Mean (SD) physician age was 52 (12.5) years (median [range] age, 54 [28-80] years). Among 121 physicians for whom specialty information was available (84.0%), 35 specialties were represented, including 45 surgeons (37.2%), 30 internists (24.8%), and 46 others (38.0%).

Original audio recordings contained a mean (SD) of 507 (296.9) words (median [range], 446 [59-1911] words) per document. The average dictation duration was 5 minutes, 46 seconds (median [range], 4 minutes, 45 seconds [21 seconds to 31 minutes, 35 seconds]). The average turnaround time was 3 hours, 37 minutes (median [range], 1 hour, 1 minute [2 minutes to 38 hours, 45 minutes]). The average physician review time was 4 days, 13 hours, 16 minutes (median [range], 23 hours, 25 minutes [0 minutes to 146 days, 4 hours, 54 minutes]).

There were 329 errors in the 33-note subset. For the 171 errors that were identified by both annotators, interannotator agreement was 71.9%. Each of the annotators failed to identify a mean (SD) of 21.7% (1.5%) of the errors that were annotated by the other. Of the errors identified by only 1 annotator, 32 errors (20.3%) pertained to clinical information; the remaining 126 (79.7%) involved minor changes to general English words. Agreement for clinical significance was 85.7%. Examples of errors of each type that were identified in this data set can be found in Table 1.

Detailed results of our error analysis are shown in Table 2 and Table 3. Errors were prevalent in original SR transcriptions, with an overall mean (SD) error rate of 7.4% (4.8%). The rate of errors decreased substantially following revision by MTs, to 0.4%. Errors were further reduced in SNs, which had an overall error rate of 0.3%. The number of notes containing at least 1 error also decreased with each processing stage. Of the 217 original SR transcriptions, 209 (96.3%) had errors. Following transcriptionist revision, this number decreased to 129 (58.1%), and by the time notes were signed, only 92 (42.4%) contained errors.

**Table 2.** Summary of Error Rates by Note Type and Processing Stage

Note Type (No.)	Note Stage	Errors, All Types				Clinically Significant Errors			
		No. of Total Errors/Total No. of Words in Criterion Standard (%)	No. of Notes With Errors/Total No. of Notes (%)	Mean Errors per Note, No.	Error Rate by Note, Median (IQR)	No. of Total Errors/Total No. of Words in Criterion Standard (%)	No. of Notes With Errors/Total No. of Notes (%)	Mean Errors per Note, No.	Error Rate by Note, Median (IQR)
Discharge summaries (75)	SR	3892/42 873 (9.1)	75/75 (100.0)	51.9	8.2 (5.2-11.7)	248/42 873 (0.6)	69/75 (90.7)	3.3	0.2 (0-0.6)
	MT	195/42 873 (0.5)	46/75 (61.3)	2.6	0.3 (0.0-0.6)	13/42 873 (0.03)	12/75 (16.0)	0.2	0
	SN	163/42 873 (0.4)	41/75 (54.7)	2.2	0.2 (0.0-0.5)	6/42 873 (0.01)	6/75 (8.0)	0.1	0
Office notes (83)	SR	2513/35 841 (7.0)	76/83 (91.6)	30.3	5.8 (3.4-8.9)	136/35 841 (0.4)	39/83 (47.0)	1.6	0 (0-0.3)
	MT	239/35 841 (0.7)	43/83 (51.8)	2.9	0.2 (0.0-0.4)	17/35 841 (0.05)	12/83 (14.5)	0.2	0
	SN	53/35 841 (0.1)	22/83 (26.5)	0.6	0.0 (0.0-0.2)	9/35 841 (0.03)	7/83 (8.4)	0.1	0
Operative notes (59)	SR	1756/31 466 (5.6)	58/59 (98.3)	29.8	4.6 (3.2-8.2)	84/31 466 (0.3)	31/59 (52.5)	1.4	0 (0-0.2)
	MT	138/31 466 (0.4)	37/59 (62.7)	2.3	0.2 (0.0-0.6)	11/31 466 (0.03)	8/59 (13.6)	0.2	0
	SN	112/31 466 (0.4)	29/59 (49.2)	1.9	0.0 (0.0-0.5)	6/31 466 (0.02)	4/59 (6.8)	0.2	0
All notes (217)	SR	8161/110 180 (7.4)	209/217 (96.3)	37.6	6.1 (3.9-10.1)	468/110 180 (0.4)	138/217 (63.6)	2.2	0 (0-0.4)
	MT	461/110 180 (0.4)	129/217 (58.1)	2.1	0.2 (0.0-0.5)	41/110 180 (0.04)	32/217 (14.7)	0.2	0
	SN	328/110 180 (0.3)	92/217 (42.4)	1.5	0.0 (0.0-0.4)	21/110 180 (0.02)	17/217 (7.8)	0.1	0

Abbreviations: IQR, interquartile range; MT, medical transcriptionist–edited notes; SN, signed notes; SR, automatic transcriptions by the speech recognition system.

**Table 3.** Error Types in Dictated Notes by Note Type and Processing Stage

Note Type (No.)	Note Stage	Total Errors, No.	No. (%)																
			Errors, General Type^a^								Errors, Semantic Type^a^								
			Deletion	Insertion	Substitution				No.	Punctuation	General English^c^	Clinical Information							
					Enunciation	Homonym	Nonsense	Other^b^				Medication	Diagnosis	Procedure	Symptom	Laboratory Test	Physical Examination	Imaging Test	Other^d^
Discharge summaries (75)	SR	3892	1395 (35.8)	1031 (26.5)	655 (16.8)	5 (0.1)	272 (7.0)	186 (4.8)	133 (3.4)	215 (5.5)	3314 (85.1)	127 (3.3)	76 (2.0)	13 (0.3)	60 (1.5)	50 (1.3)	30 (0.8)	25 (0.6)	197 (5.1)
	MT	195	87 (44.7)	36 (18.5)	35 (17.9)	0	5 (2.6)	20 (10.3)	9 (4.6)	3 (1.5)	133 (68.2)	14 (7.2)	9 (4.6)	2 (1.0)	7 (3.6)	2 (1.0)	14 (7.2)	2 (1.0)	12 (6.2)
	SN	163	74 (45.4)	29 (17.8)	29 (17.8)	0	1 (0.6)	19 (11.7)	8 (4.9)	3 (1.8)	114 (69.9)	6 (3.7)	9 (5.5)	2 (1.2)	7 (4.3)	2 (1.2)	14 (8.6)	2 (1.2)	7 (4.3)
Office notes (83)	SR	2513	875 (34.8)	549 (21.8)	608 (24.2)	10 (0.4)	178 (7.1)	128 (5.1)	41 (1.6)	124 (4.9)	2166 (86.2)	55 (2.2)	60 (2.4)	27 (1.1)	40 (1.6)	15 (0.6)	10 (0.4)	8 (0.3)	132 (5.3)
	MT	128	39 (30.5)	26 (20.3)	39 (30.5)	1 (0.8)	11 (8.6)	10 (7.8)	2 (1.6)	0	108 (84.4)	1 (0.8)	7 (5.5)	2 (1.6)	2 (1.6)	1 (0.8)	0	0	7 (5.5)
	SN	53	16 (30.2)	17 (32.1)	10 (18.9)	0	6 (11.3)	3 (5.7)	1 (1.9)	0	44 (83.0)	0	5 (9.4)	0	1 (1.9)	1 (1.9)	0	0	2 (3.8)
Operative notes (59)	SR	1756	559 (31.8)	620 (35.3)	226 (12.9)	13 (0.7)	124 (7.1)	77 (4.4)	40 (2.3)	97 (5.5)	1393 (79.3)	4 (0.2)	39 (2.2)	140 (8.0)	8 (0.5)	1 (0.1)	5 (0.3)	1 (0.1)	165 (9.4)
	MT	138	48 (34.8)	37 (26.8)	25 (18.1)	1 (0.7)	15 (10.9)	9 (6.5)	1 (0.7)	2 (1.4)	96 (69.6)	0	5 (3.6)	15 (10.9)	4 (2.9)	0	1 (0.7)	1 (0.7)	16 (11.6)
	SN	112	43 (38.4)	35 (31.3)	19 (17.0)	1 (0.9)	5 (4.5)	6 (5.4)	1 (0.9)	2 (1.8)	85 (75.9)	0	4 (3.6)	10 (8.9)	2 (1.8)	0	1 (0.9)	1 (0.9)	9 (8.0)
All notes (217)	SR	8161	2829 (34.7)	2200 (27.0)	1489 (18.2)	28 (0.3)	574 (7.0)	391 (4.8)	214 (2.6)	436 (5.3)	6873 (84.2)	186 (2.3)	175 (2.1)	180 (2.2)	108 (1.3)	66 (0.8)	45 (0.6)	34 (0.4)	494 (6.1)
	MT	461	174 (37.7)	99 (21.5)	99 (21.5)	2 (0.4)	31 (6.7)	39 (8.5)	12 (2.6)	5 (1.1)	337 (73.1)	15 (3.3)	21 (4.6)	19 (4.1)	13 (2.8)	3 (0.7)	15 (3.3)	3 (0.7)	35 (7.6)
	SN	328	133 (40.5)	81 (24.7)	58 (17.7)	1 (0.3)	12 (3.7)	28 (8.5)	10 (3.0)	5 (1.5)	243 (74.1)	6 (1.8)	18 (5.5)	12 (3.7)	10 (3.0)	3 (0.9)	15 (4.6)	3 (0.9)	18 (5.5)

Abbreviations: MT, medical transcriptionist–edited notes; SN, signed notes; SR, automatic transcriptions by the speech recognition system.

^a^ Percentages are equal to the number of errors of each type divided by the total number of errors; percentages may not sum to 100 because of rounding.

^b^ Includes suffix, prefix, dictionary, and spelling errors.

^c^ Includes general English, stop word, and date errors.

^d^ Includes patient or physician identifier, interpretation, psychological test, and ??? (unintelligible or otherwise unclassifiable) errors.

The effect of human review on note accuracy becomes more pronounced when considering just those errors that are clinically significant, rather than treating all errors as equally meaningful. Prior to human revision, 138 of 217 notes (63.6%) had at least 1 clinically significant error, with a mean (SD) of 2.2 (2.7) errors per note. After being edited by an MT, 32 notes (14.7%) had clinically significant errors, and only 17 SNs (7.8%) contained such errors. However, the proportion of errors involving clinical information increased from 15.8% to 26.9% after transcriptionist revision, although it decreased slightly to 25.9% in SNs. Similarly, the proportion of errors that were clinically significant increased from 5.7% in the original SR transcriptions to 8.9% after being edited by an MT, then decreased to 6.4% in SNs.

Table 3 shows the number and proportion of each error type across the 3 processing stages for each note type and for all notes combined. At all stages, deletion was the most prevalent general type (34.7%), followed by insertion (27.0%). The most frequent semantic type was general English. Medication was the most common clinical semantic type in the original SR transcriptions, while diagnosis was most common in the transcriptionist-edited and signed versions.

Transcriptionists made stylistic changes to 180 (82.9%) of the 217 notes and rearranged the contents of 37 notes (17.1%), usually at the request of the dictating physician. Physicians made stylistic changes to 71 notes (32.7%) and rearranged the contents of 8 notes (3.7%). Finally, there were 59 notes (27.2%) to which the signing physician added information, and 37 (17.1%) from which the physician deleted information.

The original SR transcriptions of notes created at institution A had a higher mean rate of errors compared with those created at institution B (Table 4), although not significantly, with mean error rates of 7.6% and 6.6%, respectively (difference, 1.0%; 95% CI, −0.2% to 2.8%; *P* = .10). Following human revision, however, institution A’s notes had lower error rates compared with notes from institution B, with mean error rates of 0.3% (institution A) and 0.7% (institution B) (difference, −0.3%; 95% CI, −0.63% to −0.04%; *P* = .03) after revision by an MT, and of 0.2% (institution A) and 0.6% (institution B) (difference, −0.4%; 95% CI, −0.7% to −0.2%; *P* = .003) after author review. Errors in original SR transcriptions occurred at similar frequencies for male and female physicians, with 7.5 and 7.7 mean errors per 100 words, respectively (difference, 0.2%; 95% CI, −1.2% to 1.6%; *P* = .78). A modest negative correlation was observed between age and error rate in original SR transcriptions (*r* = −0.20; 95% CI, −0.35 to −0.04; *P* = .01), with average error rates decreasing as physician age increased.

**Table 4.** Mean Error Rates Compared by Institution, Note Type, Physician Sex, and Specialty

Group 1 (No.) vs Group 2 (No.)	SR			MT			SN		
	Error Rate, Mean (SD)		*P* Value	Error Rate, Mean (SD)		*P* Value	Error Rate, Mean (SD)		*P* Value
	Group 1	Group 2		Group 1	Group 2		Group 1	Group 2	
Institution^a^									
Institution A vs institution B	7.6 (5.1)	6.6 (3.4)	.10	0.3 (0.5)	0.7 (1.0)	.03	0.2 (0.4)	0.6 (1.0)	.003
Note type									
Discharge summaries (75) vs others (142)	8.9 (4.6)	6.6 (4.7)	<.001	0.4 (0.7)	0.4 (0.6)	.51	0.4 (0.7)	0.2 (0.5)	.08
Office notes (83) vs others (134)	7.0 (5.0)	7.6 (4.7)	.33	0.3 (0.5)	0.4 (0.7)	.28	0.1 (0.3)	0.4 (0.7)	<.001
Operative notes (59) vs others (158)	6.1 (4.3)	7.9 (4.9)	.01	0.4 (0.7)	0.4 (0.9)	.72	0.4 (0.7)	0.3 (0.5)	.34
Sex^b^									
Female (69) vs male (138)	7.7 (4.8)	7.5 (4.9)	.78	0.5 (6.4)	0.4 (6.7)	.65	0.3 (0.6)	0.3 (0.6)	.96
Specialty^c^									
General, internal, or family (53) vs others (138)	8.7 (4.6)	6.9 (4.7)	.02	0.5 (0.8)	0.4 (0.6)	.60	0.4 (0.7)	0.3 (0.6)	.38
Surgery (63) vs others (128)	6.0 (4.3)	8.1 (4.7)	.002	0.4 (0.5)	0.4 (0.7)	.49	0.3 (0.5)	0.3 (0.7)	.55
Subspecialties (75) vs others (116)	7.7 (4.8)	7.2 (4.6)	.48	0.4 (0.7)	0.4 (0.6)	.93	0.3 (0.6)	0.3 (0.6)	.70

Abbreviations: MT, medical transcriptionist–edited notes; SN, signed notes; SR, automatic transcriptions by the speech recognition system.

^a^ Number omitted to preserve deidentification.

^b^ Physician sex was missing for 10 notes.

^c^ Physician specialty was missing for 26 notes.

Across all of the original SR transcriptions, discharge summaries had higher error rates than other note types (8.9% vs 6.6%; difference, 2.3%; 95% CI, 1.0%-3.6%; *P* < .001), and operative notes had lower error rates (6.1% vs 7.9%; difference, 1.8%; 95% CI, 0.4%-3.2%; *P* = .01). Likewise, notes dictated by surgeons had lower error rates than physicians of other specialties (6.0% vs 8.1%; difference, 2.2%; 95% CI, 0.8%-3.5%; *P* = .002). There was no significant difference in word counts between notes dictated by surgeons and notes dictated by physicians of other specialties; nor was there a significant difference in word counts between operative notes and other note types. No correlation was observed between word count and error rate at any stage (SR: *r* = 0.006; 95% CI, −0.13 to 0.14; *P* = .93 vs MT: *r* = 0.03; 95% CI, −0.1 to 0.2; *P* = .67 vs SN: *r* = 0.01; 95% CI, −0.1 to 0.1; *P* = .85).

## Discussion

This study is among the first, to our knowledge, to analyze errors at the different processing stages of documents created with a back-end SR system. We defined a comprehensive schema to systematically classify and analyze errors across multiple note types. The comparatively large sample and the variety of clinicians and hospitals represented increase the robustness of our findings vs those of previous studies. Of 33 studies included in 2 recent systematic reviews of SR use in health care,^5,7^ most evaluated the productivity of SR-assisted dictation compared with traditional transcription and typing. Only 16, of which 7 were conducted in the United States, reported error or accuracy rates.^20,21,22,23,24,25,26^ Many had small samples (14 included <10 clinicians) and only reviewed notes from 1 medical specialty,^27,28,29,30^ usually radiology.^20,31,32,33^ In contrast, our findings are based on dictations from 144 physicians across a wide range of clinical settings and at 2 geographically distinct institutions.

Speech recognition technology is being adopted at increasing rates at health care institutions across the country owing to its many advantages. Documentation is one of the most time-consuming parts of using EHR technology, and SR technology promises to improve documentation efficiency and save clinicians time. In back-end systems, SR software automatically converts clinicians’ dictations to text that MTs can quickly review and edit, reducing turnaround time and increasing productivity; however, it should be noted that turnaround times are typically stipulated in the contract with the transcriptionist vendor and may vary widely for this reason. Additionally, some notes remained unsigned for weeks or months, although they can still be viewed by other EHR users during this time. Many hospitals are adopting front-end dictation systems, where clinicians must review and edit their notes themselves, either as they dictate or at a later time. Clinicians face pressure to decrease documentation time and often only superficially review their notes before signing them.^9^ Fully shifting the editing responsibility from transcriptionists to clinicians may lead to increased documentation errors if clinicians are unable to adequately review their notes.

Basma et al^32^ reported that SR-generated breast imaging reports were 8 times more likely than conventionally dictated reports (23% vs 4% before adjusting confounders) to contain major errors that could affect the understanding of a report or alter patient care. Our study also identified errors involving clinical information that could have such unintended impacts. For example, we found an SN that incorrectly listed a patient as having a “grown mass” instead of a “groin mass” because of an uncorrected error in the original SR transcription. We also found evidence suggesting some clinicians may not review their notes thoroughly, if they do so at all. Transcriptionists typically mark portions of the transcription that are unintelligible in the original audio recording with blank spaces (eg, *??__??*), which the physician is then expected to fill in. However, we found 16 SNs (7.4%) that retained these marks, and in 3 instances, the missing word was discovered to be clinically significant. While additional medical record review found no evidence for the persistence of these omissions in subsequent documentation, such a risk still exists.

Although adoption of SR technology is intended to ease some of the burden of documentation, that even readily apparent pieces of information at times remain uncorrected raises concerns about whether physicians have sufficient time and resources to review their dictated notes, even to a superficial degree. As previously mentioned, a recent study in Australia reported that emergency department clinicians needed 18% more time for documentation when using SR than when using a keyboard and mouse.^4^ The authors also observed 4.3 times as many errors in SR-generated documents compared with those created with a keyboard and mouse. We observed a similar trend; the SR transcriptions we reviewed had a mean (SD) of 7.4 (4.8) errors per 100 words, while in an earlier study we found errors in typed notes at a rate of 0.45 errors per 100 words. However, SR technology is continually improving, while clinicians’ skills with and attention to keyboard and mouse documentation may not be improving at a similar rate.

In general, health information technology and the EHR have introduced a number of potential sources for error. A recent study found higher rates of errors in the EHR than in paper records, possibly attributable to EHR-specific functionality such as templates and the ability to copy and paste text.^34^ Taken together, these findings demonstrate the necessity of further studies investigating clinicians’ use of and satisfaction with SR technology, its ability to integrate with clinicians’ existing workflows, and its effect on documentation quality and efficiency compared with other documentation methods. In addition, these findings indicate a need not only for clinical quality assurance and auditing programs, but also for clinician training and education to raise awareness of these errors and strategies to reduce them.

### Limitations

The notes in our analysis were all created using the same back-end SR service. Furthermore, while larger in scale than many previous studies, our analysis was still conducted on a relatively small set of notes created in a limited number of clinical settings. As such, our findings may not be generalizable to SR-assisted documentation as a whole. Additionally, sex and specialty information was unavailable in the data sources to which we had access for 10 and 26 physicians, respectively. These missing data may limit our ability to draw conclusions about the effect these characteristics may have on error rates.

Despite the iterative testing and revision that preceded the annotation schema’s finalization, there are some additional error types we may wish to include in subsequent work. For example, the lack of a body location semantic type resulted in some confusion over how errors involving these words should be annotated, potentially leading to inconsistent annotations. In some cases, it may have been useful to divide an existing type into more granular subtypes. In particular, the stop word semantic type, which was included to distinguish short, frequently used words from other general English terms, may have inadvertently masked the true prevalence of highly specific but still commonly observed errors, such as those involving pronouns (eg, *he* or *she*) or negations.

Because of the time-intensive nature of the annotation task, we calculated interannotator agreement using only a small subset (33 of 651 [5.0%]), rather than requiring both individuals to annotate the full set of notes. This subset also included primarily notes that had been edited by MTs (26 of 33 [78.7%]), owing to the fact that errors in these notes are often more difficult to identify and may generate more disagreement.

### Future Directions

These findings lay the groundwork for many subsequent research activities. First, the developed schema can be used to annotate more notes, obtained from a wider variety of clinical domains, to create a robust corpus of errors in clinical documents created with SR technology. The benefits of such a corpus are considerable. Not only will it allow for more reliable error prevalence estimates, but it can also serve as training data for the development of an automatic error detection system. With the rapid adoption of SR in clinical settings, there is a need for automated methods based on natural language processing for identifying and correcting errors in SR-generated text. Such methods are vital to ensuring the effective use of clinicians’ time and to improving and maintaining documentation quality, all of which can, in turn, increase patient safety.

## Conclusions

Seven in 100 words in unedited clinical documents created with SR technology involve errors and 1 in 250 words contains clinically significant errors. The comparatively low error rate in signed notes highlights the crucial role of manual editing and review in the SR-assisted documentation process.

## References

[1] Poissant L, Pereira J, Tamblyn R, Kawasumi Y The impact of electronic health records on time efficiency of physicians and nurses: a systematic review. J Am Med Inform Assoc. 2005;12(5):-. 15905487 10.1197/jamia.M1700 PMC1205599

[2] Pollard SE, Neri PM, Wilcox AR, How physicians document outpatient visit notes in an electronic health record. Int J Med Inform. 2013;82(1):39-46. 22542717 10.1016/j.ijmedinf.2012.04.002

[3] Stewart B. Front-End Speech 2014: Functionality Doesn't Trump Physician Resistance. Orem, UT: KLAS; 2014 https://klasresearch.com/report/front-end-speech-2014/940. Accessed April 26, 2018.

[4] Hodgson T, Magrabi F, Coiera E Efficiency and safety of speech recognition for documentation in the electronic health record. J Am Med Inform Assoc. 2017;24(6):1127-1133. 29016971 10.1093/jamia/ocx073 PMC7651984

[5] Johnson M, Lapkin S, Long V, A systematic review of speech recognition technology in health care. BMC Med Inform Decis Mak. 2014;14:94. 25351845 10.1186/1472-6947-14-94 PMC4283090

[6] Hammana I, Lepanto L, Poder T, Bellemare C, Ly MS Speech recognition in the radiology department: a systematic review. Health Inf Manag. 2015;44(2):4-10. 26157081 10.1177/183335831504400201

[7] Hodgson T, Coiera E Risks and benefits of speech recognition for clinical documentation: a systematic review. J Am Med Inform Assoc. 2016;23(e1):e169-e179. 26578226 10.1093/jamia/ocv152 PMC4954615

[8] Safran DG, Miller W, Beckman H Organizational dimensions of relationship-centered care. theory, evidence, and practice. J Gen Intern Med. 2006;21(suppl 1):S9-S15. 16405711 10.1111/j.1525-1497.2006.00303.x PMC1484831

[9] Goss FR, Zhou L, Weiner SG Incidence of speech recognition errors in the emergency department. Int J Med Inform. 2016;93:70-73. 27435949 10.1016/j.ijmedinf.2016.05.005 PMC7263796

[10] Siegal D, Ruoff G Data as a catalyst for change: stories from the frontlines. J Healthc Risk Manag. 2015;34(3):18-25. 25630282 10.1002/jhrm.21161

[11] Ruder DB Malpractice claims analysis confirms risks in EHRs. Patient Safety and Quality Healthcare. https://www.psqh.com/analysis/malpractice-claims-analysis-confirms-risks-in-ehrs/. Published Feburary 9, 2014. Accessed April 26, 2018.

[12] Motamedi SM, Posadas-Calleja J, Straus S, The efficacy of computer-enabled discharge communication interventions: a systematic review. BMJ Qual Saf. 2011;20(5):403-415. 21262793 10.1136/bmjqs.2009.034587

[13] Rosenbloom ST, Denny JC, Xu H, Lorenzi N, Stead WW, Johnson KB Data from clinical notes: a perspective on the tension between structure and flexible documentation. J Am Med Inform Assoc. 2011;18(2):181-186. 21233086 10.1136/jamia.2010.007237 PMC3116264

[14] Davidson SJ, Zwemer FL Jr, Nathanson LA, Sable KN, Khan AN Where’s the beef? the promise and the reality of clinical documentation. Acad Emerg Med. 2004;11(11):1127-1134. 15528575 10.1197/j.aem.2004.08.004

[15] Cowan J Clinical governance and clinical documentation: still a long way to go? Clin Perform Qual Health Care. 2000;8(3):179-182. 11185832

[16] von Elm E, Altman DG, Egger M, Pocock SJ, Gøtzsche PC, Vandenbroucke JP; STROBE Initiative The Strengthening the Reporting of Observational Studies in Epidemiology (STROBE) statement: guidelines for reporting observational studies. PLoS Med. 2007;4(10):e296. 17941714 10.1371/journal.pmed.0040296 PMC2020495

[17] Ranks NL Webmaster Tools. Stopwords. https://www.ranks.nl/stopwords. Accessed April 26, 2018.

[18] Ogren PV Knowtator: a Protégé plug-in for annotated corpus construction. In: Proceedings of the 2006 Conference of the North American Chapter of the Association for Computational Linguistics on Human Language Technology; June 4-9, 2006; New York, NY. doi:10.3115/1225785.1225791

[19] Dawson L, Johnson M, Suominen H, A usability framework for speech recognition technologies in clinical handover: a pre-implementation study. J Med Syst. 2014;38(6):56. 24827759 10.1007/s10916-014-0056-7

[20] Quint LE, Quint DJ, Myles JD Frequency and spectrum of errors in final radiology reports generated with automatic speech recognition technology. J Am Coll Radiol. 2008;5(12):1196-1199. 19027683 10.1016/j.jacr.2008.07.005

[21] Zick RG, Olsen J Voice recognition software versus a traditional transcription service for physician charting in the ED. Am J Emerg Med. 2001;19(4):295-298. 11447517 10.1053/ajem.2001.24487

[22] Pezzullo JA, Tung GA, Rogg JM, Davis LM, Brody JM, Mayo-Smith WW Voice recognition dictation: radiologist as transcriptionist. J Digit Imaging. 2008;21(4):384-389. 17554582 10.1007/s10278-007-9039-2 PMC3043849

[23] Kanal KM, Hangiandreou NJ, Sykes AM, Initial evaluation of a continuous speech recognition program for radiology. J Digit Imaging. 2001;14(1):30-37. 11310913 10.1007/s10278-001-0022-z PMC3489193

[24] Zemmel NJ, Park SM, Maurer EJ, Leslie LF, Edlich RF Evaluation of VoiceType Dictation for Windows for the radiologist. Med Prog Technol. 1996-1997;21(4):177-180. 9110274

[25] Ramaswamy MR, Chaljub G, Esch O, Fanning DD, vanSonnenberg E Continuous speech recognition in MR imaging reporting: advantages, disadvantages, and impact. Am J Roentgenol. 2000;174(3):617-622. 10701598 10.2214/ajr.174.3.1740617

[26] Smith NT, Brien RA, Pettus DC, Jones BR, Quinn ML, Sarnat A Recognition accuracy with a voice-recognition system designed for anesthesia record keeping. J Clin Monit. 1990;6(4):299-306. 2230859 10.1007/BF02842489

[27] Issenman RM, Jaffer IH Use of voice recognition software in an outpatient pediatric specialty practice. Pediatrics. 2004;114(3):e290-e293. 15342888 10.1542/peds.2003-0724-L

[28] Ilgner J, Düwel P, Westhofen M Free-text data entry by speech recognition software and its impact on clinical routine. Ear Nose Throat J. 2006;85(8):523-527. 16999060

[29] Yuhaniak Irwin J, Fernando S, Schleyer T, Spallek H Speech recognition in dental software systems: features and functionality. Stud Health Technol Inform. 2007;129(Pt 2):1127-1131. 17911891

[30] Al-Aynati MM, Chorneyko KA Comparison of voice-automated transcription and human transcription in generating pathology reports. Arch Pathol Lab Med. 2003;127(6):721-725. 12741898 10.5858/2003-127-721-COVTAH

[31] Voll K, Atkins S, Forster B Improving the utility of speech recognition through error detection. J Digit Imaging. 2008;21(4):371-377. 17387554 10.1007/s10278-007-9034-7 PMC3043851

[32] Basma S, Lord B, Jacks LM, Rizk M, Scaranelo AM Error rates in breast imaging reports: comparison of automatic speech recognition and dictation transcription. AJR Am J Roentgenol. 2011;197(4):923-927. 21940580 10.2214/AJR.11.6691

[33] Vorbeck F, Ba-Ssalamah A, Kettenbach J, Huebsch P Report generation using digital speech recognition in radiology. Eur Radiol. 2000;10(12):1976-1982. 11305581 10.1007/s003300000459

[34] Yadav S, Kazanji N, Narayan KC, Comparison of accuracy of physical examination findings in initial progress notes between paper charts and a newly implemented electronic health record. J Am Med Inform Assoc. 2017;24(1):140-144. 27357831 10.1093/jamia/ocw067 PMC7654088

